# Validation of AD8-Philippines (AD8-P): A Brief Informant-Based Questionnaire for Dementia Screening in the Philippines

**DOI:** 10.1155/2021/7750235

**Published:** 2021-10-31

**Authors:** Jacqueline C. Dominguez, Ma. Fe P. de Guzman, Ma. Lourdes C. Joson, Krizelle Fowler, Boots P. Natividad, Precy S. Cruz, Jose Leo Jiloca, Primitivo B. Mactal, Jayvee Dyne Dominguez, Jeffrey Domingo, Jhozel Kim Dominguez-Awao, Macario Reandelar, Jem R. Javier, ThienKieuThi Phung, John C. Morris, James E. Galvin

**Affiliations:** ^1^Institute for Neurosciences, St. Luke's Medical Center, Quezon City 1102, Philippines; ^2^Institute for Dementia Care Asia, Quezon City 1102, Philippines; ^3^Dementia Society of the Philippines, Manila City 1008, Philippines; ^4^Research and Biotechnology Division, St. Luke's Medical Center, Quezon City 1102, Philippines; ^5^Department of Neuroscience and Behavioral Medicine, Faculty of Medicine and Surgery, University of Santo Tomas, Manila 1008, Philippines; ^6^Geriatric Center, St. Luke's Medical Center, Quezon City 1102, Philippines; ^7^St. Luke's College of Medicine William H. Quasha Memorial, Quezon City 1102, Philippines; ^8^St. Louis University College of Medicine, Baguio City 2600, Philippines; ^9^Department of Linguistics, College of Social Sciences and Philosophy, University of the Philippines, Quezon City 1100, Philippines; ^10^Department of Neurology, Danish Dementia Research Center, Rigshospitalet, University of Copenhagen, Copenhagen 2100, Denmark; ^11^Department of Neurology, Washington University School of Medicine, St. Louis, MO, USA; ^12^Comprehensive Center for Brain Health, Department of Neurology, University of Miami Miller School of Medicine, USA

## Abstract

**Aim:**

This study was aimed at validating the Filipino version of AD8 (AD8-P).

**Methods:**

Community-dwelling Filipino older persons aged ≥60 years, together with their informants, participated in this study. Psychologists independently interviewed the informants with AD8-P and administered the Filipino-validated Mini-Mental State Examination (MMSE-P) and Montreal Cognitive Assessment (MoCA-P) to the older persons. Neurologists and geriatrician conducted physical and neurological examination and Clinical Dementia Rating™ (CDR™) to determine cognitive diagnosis and were blinded with the results of AD8-P. Dementia was diagnosed based on DSM-IV-TR criteria. AD8-P discriminatory ability to screen for dementia was evaluated according to DSM-IV-TR diagnostic criteria for dementia.

**Results:**

A total of 366 community-dwelling Filipino older persons aged ≥60 years, 213 with normal cognition and 153 with dementia, and their informants were included in this study. Majority (90%) were at the mildest stage of dementia. Area under the receiver-operating-characteristic curve (AUROC) for AD8-P was 0.94 (95% CI 0.92 to 0.96), demonstrating excellent overall predictive power to screen for dementia. The optimal AD8-P cut-off score with best balance sensitivity (91.5%) and specificity (77.9%) was ≥3.

**Conclusion:**

AD8-P demonstrated good psychometric properties to screen for dementia, even at the earliest stage of cognitive decline.

## 1. Introduction

Dementia is a major cause of disability and dependency among older adults worldwide. The number of older persons living with dementia is steadily increasing and projected to reach as high as 131.5 million by 2050 [1]. In the Philippines, the prevalence of dementia was found to be 10.6% among older Filipino adults aged 60 and above [2], a proportion which is higher than the estimated 7.6% prevalence for South East Asia [1]. Despite this growing public health problem, screening and detection for dementia remain a major challenge. High prevalence of undetected dementia in the community is estimated to be 93% in Asia and between 43 and 71% in Europe and North America [3]. The causes of missed or delayed diagnosis for dementia include case complexity, resource constraints, lack of awareness, and lack of culture-sensitive and education-fair instruments for dementia screening [4]. Commonly used screening instruments for dementia in the Philippines include the Filipino-validated Mini-Mental State Examination (MMSE-P) [5] and the Montreal Cognitive Assessment (MoCA-P) [6, 7]. Both tests are strictly standardized and require training to administer and interpret. Moreover, the scores on both tests are strongly influenced by educational and other sociocultural factors [8, 9].

In the context of Asian countries where extended family structure still prevails, informant-based assessments have been demonstrated to be superior to direct cognitive testing in screening for dementia and detecting early signs of cognitive impairment [10, 11]. The consensus today is that patient- and informant-based questionnaire should be combined to enhance screening and diagnostic accuracy [12]. Informant-based assessments minimize biases such as practice effects, low education, and sociocultural influences, which confound the interpretation of direct cognitive assessments [13]. The most commonly used research assessments that incorporate information from informants to evaluate patients' cognitive ability are the Clinical Dementia Rating™ (CDR™) and the Informant Questionnaire for Cognitive Decline in the Elderly (IQCODE) [14, 15]. Both assessments have demonstrated good psychometric properties to distinguish between dementia and normal cognition [14–16]. The IQCODE has been validated in developing countries with high rates of illiteracy, demonstrating excellent discriminating power to screen for dementia in this context [17, 18]. However, both instruments are not suitable to be used for dementia screening in the community in the Philippines. The CDR is lengthy and complex to administer, a task usually done by an experienced clinician, usually a dementia specialist. The IQCODE requires the informant to rate the cognitive function of the older person using five graded categories of change (much improved, somewhat improved, no change, somewhat worse, and much worse). The differences between these categories have been found to be indistinguishable for Filipinos. A study to validate the IQCODE in Tagalog demonstrated that the participants found it impossible to quantify the degree of change between “much” and “somewhat” (Dominguez J, data unpublished). Further, the IQCODE may not discriminate normal aging from mild cognitive impairment [19]. The (AD8™) is a brief informant-based screening instrument for dementia developed by the Alzheimer's Disease Research Center, Washington University in St. Louis [20, 21]. Compared with other informant-based assessments, AD8 has been shown to be more sensitive to capture the early stages of dementia [19], regardless of the etiologies [20, 21], and corresponds to Alzheimer biomarkers [22]. The AD8 is designed to capture a decline from previous levels for an individual's cognitive and functional performance (intraindividual change), which is the sine qua non for dementia detection; the MMSE and MoCA cannot capture such decline unless administered longitudinally. Hence, the AD8 may offer this advantage over those instruments for dementia screening. In addition, all of the AD8 questions come from the informant interview of the CDR and AD8 is considered an abbreviated CDR interview; thus, the correspondence between the AD8 and the CDR should be very high. The AD8 has been validated with molecular biomarkers and neuropathologic diagnosis of Alzheimer's disease facilitating earlier and more accurate AD diagnosis in a variety of care settings [23].

The administration of AD8 requires minimal training and can be easily conducted in person or through phone calls within three minutes [20], making it particularly suitable for dementia screening in the community setting and primary care. The answers are simple (yes, no, and not applicable) and can also be self-administered by the informant [24]. In the absence of a reliable informant, the AD8 may be asked of the participant to gain an understanding of their perception of cognitive status [24]. The AD8 rates decline that can be ascribed to the cognitive loss but not due to other causes (e.g., significant visual impairment limits the ability to drive would not merit a “yes” response on the AD8).

Therefore, this study was aimed at validating the Filipino version of the AD8 (AD8–P) in screening for dementia among community-dwelling Filipino older adults.

## 2. Materials and Methods

### 2.1. Study Design and Population

Participants were community-dwelling Filipino older adults enrolled in the Marikina Memory and Aging Project (MMAP), which was a large population-based longitudinal study of dementia epidemiology in the community [2]. The cohort was established in 2011-2012 and consisted of a random sample of 1,367 participants ≥ 60 years old who were selected from the senior citizen registry in the city of Marikina [2]. We established the prevalence of dementia and cognitive impairment nondementia (CIND) in this cohort in 2011 and followed them up in 2016-2017 (5-year follow-up), when there were 831 (60.8%) participants left in the cohort. The study population for this validation study was selected from this follow-up cohort. We carried out comprehensive cognitive assessments of the whole cohort to identify persons with dementia and normal cognition. The study was approved by the St. Luke's Institutional Ethics Review Committee (CT-14064).

### 2.2. Eligibility Criteria

The eligibility criteria were as follows: (1) community-dwelling Filipinos aged ≥60 years old, (2) diagnosed with dementia according to DSM-IV TR criteria [25] and CDR ≥ 0.5 or with normal cognition (CDR = 0) [15], (3) were fluent in Tagalog, and (4) had an available informant. An informant was defined as the person who knew the participant well or coresides with the participant for at least a year, such as a relative, a friend, a neighbor, or a caregiver.

### 2.3. Translation of AD8–P

To construct the Filipino version of AD8 (AD8–P), a direct translation of the original AD8 was carried out by two Filipino bilingual experts, a neurologist (SM) and a psychologist (PM). English and Filipino are the two main official languages of the Philippines, a country where 170 different languages are spoken. The translation was reviewed and backtranslated by another psychologist and a specialist in linguistics (JJ). The Filipino version was subsequently reviewed by multidisciplinary committee from Dementia Society of the Philippines composed of neurologists, geriatricians, psychologists, nurses, and linguists led by SM and PC to evaluate the semantic and conceptual equivalence of each item in the AD8 questionnaire. The translation and backtranslation underwent 3 iterations until the final version. Some examples in the original English version were modified to be relevant to the Filipino context.

In question 4, regarding trouble learning how to use a tool, appliance, or gadget, the “VCR” example was changed to “VCD,” which was a more relevant appliance in a Filipino household at that time. Likewise, use of computer and microwave amongst the Filipino elderly was not common; thus, these were replaced with cellular phone, television, or karaoke.

The translated AD8–P questionnaire was administered to five Filipino older adults to examine the face validity of the questionnaire and was found easy to understand and useful by the informants. The translated work was approved by the author of the AD8, Dr. James E. Galvin (JEG). Washington University in St. Louis, MO, granted license to the Dementia Society of the Philippines to lead the translation and validation.

### 2.4. Evaluation Procedure

Two community health workers provided by the city government of Marikina visited the older persons at home and coordinated their scheduled visits at the research site accompanied by their informants. The evaluation was carried out by well-trained psychologists (MDG, HSS, RLC, and CP), a geriatrician (JLJ), and neurologists (JCD, MCJ). AD8-P was administered by trained medical interns (JDD, JD, and JKD). Both the selected older persons and their informant participated in the assessments.

The psychologists independently administered the following assessments:

(A) To the informants
  - Filipino version of AD8 (AD8-P): similar to the original English version [20], the AD8-P consists of 8 items that inquire into cognitive domains such as memory (frequent problems in memory, repetition, and remembering appointments), temporal orientation (trouble with month or year), judgment (making decision, handling finances), and function (reduced interest in activities, use of appliances). The score ranges from 0 to 8, with higher scores indicating worse cognitive function
  - Lawton's Scale for Instrumental Activities of Daily Living (IADL) [26]: it is a functional scale to assess eight complex activities of daily living for the older person. The score ranges from 0 to 32, lower scores indicating better functional levels
(B) To the older persons
  - The Filipino-validated Mini-Mental State Examination (MMSE–P) [5]: MMSE measures orientation, attention, language, memory, and constructional praxis. In the Filipino MMSE-P, the serial subtraction was replaced with spelling backward a five-letter word (MUNDO or world in English). The score ranges from 0 to 30; lower score indicates worse cognitive function. The MMSE-P has demonstrated good psychometric properties to screen for dementia with a sensitivity of 85% and a specificity of 86% at the cut-off score of ≤23 [5]
  - The Filipino-validated Montreal Cognitive Assessment (MoCA–P) [6, 7]: MoCA-P was designed as a screening instrument for mild cognitive dysfunction. It assesses different cognitive domains: attention and concentration, executive functions, memory, language, visuospatial functions, conceptual thinking, calculations, and orientation. The score ranges from 0 to 30, with lower scores indicating worse cognitive function. A MoCA-P cut-off of ≤20 for dementia demonstrated psychometric property of 84% sensitivity and 72% specificity [6]
  - Geriatric Depression Scale (GDS) [27]: it is the most common scale currently used to measure depression in older adults. We used the brief version, the 15-item scale. A score of 5 or more indicates possible depression

The neurologists performed the following assessments:

- Clinical Dementia Rating™ [15]: the neurologist rated the CDR based on semistructured interview responses from the older persons and their informants. The CDR Sum of Boxes score is a total score ranging from 0 to 18 based on the sum of 6 domain scores (orientation, judgment and problem solving, memory, home and hobbies, personal care, and community affairs). Each domain is rated in five categories: normal (0), questionable or very mild dementia (0.5), mild dementia (1), moderate dementia (2), and severe dementia (3). These domains are then combined into a global CDR that ranges from 0 to 3
- Physical and neurological examinations

### 2.5. Case Ascertainment

The neurologist (JCD, MJC) and geriatrician (JLJ) conducted medical history and physical examinations, CDR [15], and synthesized results of neuropsychological assessments, while being blinded to the result of AD8-P. All assessment data were reviewed by the physician, and they consulted with each other as necessary to reach a consensus on dementia diagnosis. Dementia was diagnosed according to DSM-IV TR criteria [25] and with CDR scores ≥ 0.5. Participants were classified as having normal cognition and no depression if they had a CDR = 0, did not fulfill DSM-IV TR criteria for dementia, and scored ≤4 on GDS.

### 2.6. Statistical Analysis

Data analysis was performed using SPSS (version 23; IBM). Statistical tests were 2-tailed at significant level set at *p* ≤ 0.05. The overall predictive ability of the AD8-P was evaluated using AUROC (area under the receiver operating characteristic curve, sensitivity plotted against 1 − specificity). The AUROC was used to choose the cut-off point with the best balance of sensitivity and specificity for the AD8-P. At the chosen cut-off point, the following parameters were estimated for the AD8-P: sensitivity, specificity, positive predictive value (PPV), negative predictive value (NPV), and Youden's index ( (sensitivity + specificity) − 1, summarizing sensitivity and specificity in a single measure). Chi-square test (or Fisher exact test when ≥20 cells have expected count < 5) to compare proportions. The Student *t*-test for independent samples was used to compare the means of age, education, and assessment scores between groups. To determine whether the older persons' characteristics (age, gender, and education) and the informants' characteristics (gender, age, education, and relationship to the older persons) affect the AD8-P scores, Student's *t*-test and one-way ANOVA were used to compare mean scores among the normative (reference) group. Pearson correlation coefficient (*r*) was used to assess relationships between continuous variables.

## 3. Results

A total of 366 older persons and their informants were included in the study. The older persons' mean age was 73.4 (standard deviation or SD = 6.97) years old (range 63-103 years), and the mean educational attainment was 9.1 (SD = 4.12) years (range 0 to 18 years). Majority of the older persons (69.9%) and their informants (63.7%) were women. The informants were mainly children (44.0%), spouses (38.9%), grandchildren (6.0%), and others such as daughters in law, siblings, neighbors, or close friends (11.1%). Among the 366 older persons, 153 (41.8%) had dementia and 213 (58.2%) had normal cognition without depression (Table 1). Among those with dementia, 106 (69.3%) had very mild dementia (CDR = 0.5), 32 (20.9%) had mild dementia (CDR = 1), 7 (4.6%) had moderate dementia (CDR = 2), and 8 (5.2%) had severe dementia (CDR = 3). Compared with those who were cognitively intact, older persons with dementia in this study had significantly higher age, lower education, and poorer performance on all neuropsychological and functional assessments (AD8, CDR-SB, MMSE-P, MoCA-P, and IADL, *p* < 0.001, Table 1). For each AD8-P item, the group of older persons with dementia was rated significantly worse than those with normal cognition by the informants (Table 2).

**Figure 1:**
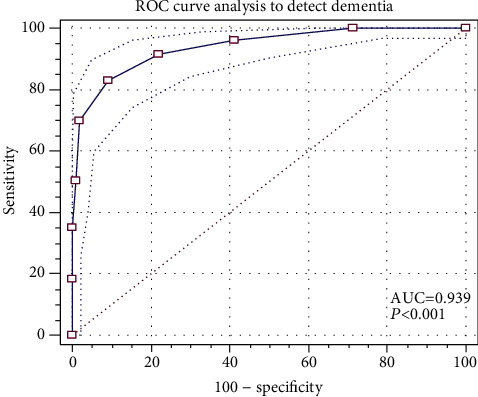
Receiver operating curve (ROC) of AD8-P to detect dementia.

The AD8-P demonstrated good internal consistency with a Cronbach alpha (*α*) of 0.82 (95% confidence interval or CI, 0.79 to 0.85). AD8-P scores were strongly correlated with scores from CDR-SB, MoCA-P, MMSE-P, and IADL (Table 3). The AUROC for the AD8-P was 0.94 (95% CI, 0.92 to 0.96), demonstrating excellent overall predictive power to distinguish between normal cognition (CDR 0) and dementia (Figure 1). A cut-off score of ≥4 has a good sensitivity (83.0%) and an excellent specificity (90.6%), whereas a cut-off score of ≥3 has an excellent sensitivity (91.5%) and a reasonable specificity (77.9%). Since AD8-P was validated as a screening instrument for dementia, it is best to use the cut-off score with the highest sensitivity (Table 4).

AD8-P score is not affected by age, education, and gender of the older persons (Table 5). Concerning informant characteristics, AD8-P score is not affected by age and education of the informants and type of relationship to the older persons. However, female informants significantly rated higher score than male informant (Table 5). Subsequently, we did a sensitivity analysis of AUROC stratified by gender and found the AUROC was comparable for male and female informants, being 0.96 (95% CI 0.92 to 0.99) for male and 0.91 (95% CI 0.87 to 0.95) for female.

## 4. Discussions

We successfully validated a Filipino version of AD8 which is a brief informant-based questionnaire for dementia screening in the community. It has high internal consistency and strong correlation with other cognitive assessments such as the CDR-SB, MMSE, and MoCA, as well as functional assessment such as the IADL. Compared to the MMSE-P and MoCA-P [5, 6], AD8-P has better psychometric properties. At the cut-off point of ≥3, AD8-P is more sensitive to detect dementia while maintaining similar specificity to MMSE-P and MoCA-P. This may be due to the fact that the AD8-P examines intraindividual decline rather than interindividual performance compared to a normative value. At the cut-off point of ≥4, AD8-P has comparable sensitivity to MMSE-P and MoCA-P but with much higher specificity. Furthermore, AD8-P score is not affected by the older persons' age, gender, and education level, which makes AD8-P particularity suitable for dementia screening among older populations with low education. To our knowledge, this is the first study to examine whether informants' characteristics affect AD8-P score. We found that AD8-P is not affected by age and education level of the informant and the type of relationship the informant has with the older person. However, gender of the informant, particularly being female, was associated with significantly higher AD8-P score. This may be because women are usually the caregivers and more aware of the subtle changes in the older persons' cognitive function. Nonetheless, excellent overall predictive ability of the AD8-P was not affected by the gender of the informants, since the AUROC was comparable for both male and female informants.

Majority of the dementia cases (90%) in this study was in the earliest stages of dementia (very mild to mild). The distribution of dementia stages among the cases identified for this validation study reflects the spectrum of dementia severity detected by dementia screening in the community, as we screened an entire community-based cohort for case finding. Thus, AD8-P is a very good instrument for early detection of dementia in the community. It is simple and brief, does not require much training, and is well accepted by informants. The results of this validation study have shown that AD8-P is particularly suitable to be used by frontline community health workers and primary care health professionals to screen for dementia in the Philippines.

We recognize the spectrum of cognitive impairment spanning from normal aging to subjective cognitive impairment to mild cognitive impairment and eventually dementia diagnosis; however, one limitation of our study is that we only analyzed the two ends of the spectrum: cognitively normal individuals and patients living with dementia. The authors plan to do a longitudinal study to follow up our cohort and include in the analysis all the clinical stages of cognitive impairment. Another possible limitation is that while frontotemporal dementia (FTD) has been studied in the context of other dementias, future studies could specifically examine the properties of the AD8 in FTD.

The optimum cut-off points for AD8-P in this study are between 3 and 4, which is higher than the recommended cut-off point ≥ 2 in the original English version of the AD8 used in the US [20, 21], which was subsequently supported through validation studies in industrialized Asian countries such as Japan, Taiwan, and South Korea with fairly well-educated study population [28–30]. However, in a Brazilian study population with lower education and mixed ethnicities, they found the same cut-off point of ≥3 as in our study [31]. The reasons given for higher cut-off point were lower education and lower socioeconomic status. It is out of context to ask the older persons if they can learn how to operate high-tech electronics and gadgets when these items are not available in their homes. The same reason may apply to the Philippines, a low middle-income country where about one-quarter of the population live in poverty and modern appliances such as microwave and computer are not common items in many households. As we can see in Table 2, informants reported problems with using appliances and devices for about one-third of the cognitively intact older persons. Nevertheless, in other industrialized countries such as Singapore and Spain, they also found the same cut-off point of ≥3 [32, 33]. Regardless of cut-off points, the sensitivity and specificity reported for AD8 in other countries are comparable to our current findings, demonstrating its universal validity to screen for dementia across cultures and languages. However, it is recommended to validate AD8 in the specific language and culture to identify the optimal cut-off point for that specific population.

## 5. Conclusion

In conclusion, the AD8-P is a valid brief screening tool for dementia in the community. It can be recommended for widespread use to facilitate early detection of dementia in the Philippines.

## Figures and Tables

**Table 1:** Characteristics of study participants.

	Normal cognition (*N* = 213)	Dementia (*N* = 153)	*p* value
Older persons' characteristics			
Gender, *n* (%)			
Male	70 (32.9)	40 (26.1)	0.17
Female	143 (67.1)	113 (73.9)	
Age; M (SD)	70.9 (5.2)	76.9 (7.7)	<0.001
Age groups; *n* (%)			
60-69 years old	100 (47.2)	31 (20.3)	<0.001
70-79 years old	96 (45.3)	65 (40.4)	
≥80 years old	16 (7.5)	57 (37.3)	
Education; *M* (SD)	10.8 (3.4)	6.9 (4.0)	<0.001
Level of education; *n* (%)			
No formal education (0 year)	1 (0.5)	11 (7.2)	<0.001
Primary education (1-6 years)	35 (16.4)	85 (55.6)	
Secondary education (7-10 years)	73 (34.3)	30 (19.6)	
Tertiary education (≥11 years)	104 (48.8)	27 (17.6)	
Informants' characteristics			
Relationship to the older person, *n* (%)			
Spouse	101 (47.4)	43 (28.1)	<0.005
Child	75 (35.2)	84 (54.9)	
Grandchild	10 (4.7)	12 (7.8)	
Others	27 (12.7)	14 (9.2)	
Gender, *n* (%)			
Male	84 (39.4)	50 (32.7)	0.23
Female	129 (60.6)	103 (67.3)	
Age; *M* (SD)	55.6 (17.0)	51.7 (16.3)	0.05
Age groups; *n* (%)			
18-24 years old (young adult)	11 (5.2)	7 (4.6)	0.02
25-64 years old (midadult)	111 (52.1)	104 (68.0)	
≥65 years old (older adult)	91 (42.7)	42 (27.4)	
Education; *M* (SD)	11.3 (3.2)	10.1 (3.6)	0.01
Level of education; *n* (%)			
No formal education (0 year)	0 (0.0)	3 (2.0)	0.01
Primary education (1-6 years)	34 (16.0)	31 (20.3)	
Secondary education (7-10 years)	55 (25.8)	60 (39.2)	
Tertiary education (≥11 years)	124 (58.2)	59 (38.6)	
Clinical assessments; *M* (SD)			
CDR-SB	0.0 (0.0)	4 (3.6)	<0.001
MMSE-P	27.9 (1.6)	19.5 (7.4)	<0.001
MoCA-P	24.4 (2.4)	11.9 (6.3)	<0.001
Lawton's IADL	8.7 (1.7)	16.7 (7.6)	<0.001

AD8-P: Filipino version of AD8; CDR–SB: Clinical Dementia Rating Scale–Sum of Boxes; MMSE–P: Filipino-validated Mini-Mental State Examination (MMSE-P); MoCA–P: Filipino-validated Montreal Cognitive Assessment; Lawton's IADL: Lawton's Instrumental Activities of Daily Living; *M*: mean; SD: standard deviation; *n*: frequency.

**Table 2:** Comparison between the groups of older persons with normal cognition and dementia for each item of the AD8-P.

AD8-P questions (reported yes by informants)	Normal (*n* = 213) *n* (%)	Dementia (n = 153) *n* (%)	*p* value
Item 1: problems with judgment	28 (13.1)	96 (62.7)	<0.001
Item 2: reduced interests and hobbies	46 (21.6)	105 (68.6)	<0.001
Item 3: repetition	36 (16.9)	83 (54.6)	<0.001
Item 4: use of appliances or devices	72 (33.8)	134 (88.2)	<0.001
Item 5: temporal orientation	39 (18.3)	116 (75.8)	<0.001
Item 6: handling finances	7 (3.3)	90 (58.8)	<0.001
Item 7: remembering appointments	29 (13.6)	96 (62.7)	<0.001
Item 8: consistent memory problems	56 (26.3)	113 (74.3)	<0.001

AD8–P: Filipino version of the AD8; *n*: frequency.

**Table 3:** Correlation of AD8–P scores with other cognitive and functional assessments.

Clinical assessments	Correlation coefficients	*p* value
CDR-SB	0.60	<0.001
MMSE-P	-0.60	<0.001
MoCA-P	-0.71	<0.001
Lawton's IADL	0.61	<0.001

AD8-P: Filipino version of AD8; CDR–SB: Clinical Dementia Rating Scale–Sum of Boxes; MMSE–P: Filipino-validated Mini-Mental State Examination (MMSE-P); MoCA–P: Filipino-validated Montreal Cognitive Assessment; Lawton's IADL: Lawton's Instrumental Activities of Daily Living.

**Table 4:** Psychometric properties of AD8-P at different cut-off points.

Cut-off	Sensitivity	Specificity	Youden index	PPV	NPV	Correctly identified
≥2 points	96.1 (0.91-0.98)	42.1 (0.34-0.50)	0.38	62.6 (0.56-0.69)	91.4 (0.82-0.96)	57.7 (0.53-0.63)
≥3 points	91.5 (0.85-0.95)	77.9 (0.72-0.83)	0.69	74.9 (0.68-0.81)	92.7 (0.87-0.96)	83.6 (0.79-0.87)
≥4 points	83.0 (0.76-0.88)	90.6 (0.86-0.94)	0.74	86.4 (0.86-0.80)	88.1 (0.83-0.92)	87.4 (0.84-0.91)

All values except Youden's index are percentages with 95% confidence interval in parentheses. PPV: positive predictive value; NPV: negative predictive value; AD8–P: Filipino version of the AD8.

**Table 5:** Comparisons of mean AD8-P scores stratified by the older persons' and informants' characteristics.

Participants' characteristics	AD8-P scores *M* (SD)	*p* value
Older persons' characteristics		
Gender		
Male	1.7 (1.5)	0.16
Female	1.4 (1.3)	
Age groups		
60-69 years old	1.3 (1.2)	0.12
70-79 years old	1.7 (1.4)	
≥80 years old	1.7 (1.5)	
Education groups^∗^		
Primary education (1-6 years)	1.4 (1.4)	0.36
Secondary education (7-10 years)	1.4 (1.3)	
Tertiary education (≥11 years)	1.8 (1.4)	
Informants' characteristics		
Gender		
Male	1.0 (1.0)	0.001
Female	1.7 (1.5)	
Relationship to the older person		
Spouse	1.7 (1.4)	0.25
Child	1.2 (1.3)	
Grandchild	1.4 (1.1)	
Others	1.4 (1.4)	
Age groups		
18-24 years old (young adult)	1.2 (1.5)	0.35
25-64 years old (mid-adult)	1.3 (1.3)	
≥65 years old (older adult)	1.6 (1.4)	
Education groups^∗^		
Primary education (1-6 years)	1.3 (1.2)	0.71
Secondary education (7-10 years)	1.4 (1.1)	
Tertiary education (≥11 years)	1.5 (1.6)	

^∗^No formal education was not included in the analysis because it has fewer than two cases for group with normal cognition. AD8–P: Filipino version of the AD8; *M*: mean; SD: standard deviation.

## Data Availability

The technical appendix, statistical code, and dataset used to support the findings in this study will be made available from the corresponding author upon request.
